# 

**Published:** 1914-08-15

**Authors:** 


### CONTENTS.

PAGE

**Linking Up New Hospital Units** ... ... ... 537

**The War and the Poor-Law Infirmaries** ... 538

**Hospital and Institutional News** ... ... ... 539

Gift of a Base Hospital to the Queen: Arrangements and Staff—Panic Offers of Private Houses—A Post which no One is Asked to Fill—The London Hospital Staff who have Left for the Front—Duchess of Westminster's War Hospital—London Hospitals' Offer to War Office and Admiralty—Hospital Preparations by Private Generosity—Hospital Advice on the Conversion of Houses—The Queen and Hospital Linen Guilds—Convalescent Homes for Wounded Sailors—Cottage Hospitals and the Wounded—The Patriotism of the Hull Guardians—Effects of the War on Private Practice—The Crystal Palace as a Hospital—The First Accepted Hospital Ships—The Stigma on Birmingham Attendants—End of the Blackpool Hospital Deadlock: The Board Resign—Guy's Hospital and Defective Recruits—Overcrowding at a Manchester Infirmary—Women's Competition for Secretarial Post—The Infection of Non-phthisical Patients—The Ending of the Barry Hospital Dispute—The Final Solution of Workhouse Nursing—The Doctors' Wives Association—Germany and the Drug Market.

A STATE OF WAR.

**The Country and the Care of the Sick and Wounded** ... ... ... ... ... ... 545
What the Country is Doing.

**London Hospitals: Departures and Plans** ... 546

The City of London.

**Emergency Work in the Provinces** ... ... 547

Extemporised Hospitals.

PAGE

**The East Coast and the War** ... ... ... 549
A View of the Hospital Arrangements.

**Preparations in Scotland** ... ... ... ... 550
The Arrangements at Edinburgh and Dundee.

**Hospitals and the War** ... ... ... ... 551
The Problem for Hospital Staffs and the Colleges.

**Lord Haldane's Territorial Hospitals and Nursing Service** ... ... ... ... ... 553

**London Panel Committee and the War** ... 554
Free Treatment for those Affected.

**The Imperial Forces and National Insurance** 555
The Position of Sailors and Soldiers.

**Buildings Contemplated** ... ... ... ... 556

**The Editor's Letter-Box** ... ... ... ... 557
St. Mark's Hospital—Poor-Law Infirmaries and the War.

**A Newcastle Hospital Pioneer** ... ... ... 557

**Minor Hospital War News** ... ... ... ... 557

**Round the World** ... ... ... ... ... 558

Fellow-Workers and their Doings.

**Institutional Organisation in America** ... 559
Conundrums about Hospital Efficiency.

**Medical Appointments** ... ... ... ... 559

**The Profession of Medicine** ... ... ... 560
Quacks and the Bounds of Human Credulity.
Dr. Addison's New Post.

**The Institutional Worker** ... ... (Suppl.) 1
Dinner Tins for a Fever Sanatorium.
Points in Institutional Housekeeping.
Questions for August and September.
Questions Invited.
Practical Matters.
Employment and Training.
Acknowledgments.

